# Supplementary material for: seqgra: principled selection of neural network architectures for genomics prediction tasks
Source: Bioinformatics. 2022 Feb 22;38(9):2381–8. doi: 10.1093/bioinformatics/btac101 (PMC9048673; doi:10.1093/bioinformatics/btac101)
Supplement: btac101_Supplementary_Data [file btac101_supplementary_data.pdf]

Supplementary Material

seqgra: Principled Selection of Neural Network Architectures for Genomics Prediction Tasks

Konstantin Krismer[1,2], Jennifer Hammelman[1,3] and David K. Gifford[1,2,3,4,*]

[1]Computer Science and Artificial Intelligence Laboratory, Massachusetts Institute of Technology, 32 Vassar Street, Cambridge, MA 02139, USA
[2]Department of Biological Engineering, Massachusetts Institute of Technology, 77 Massachusetts Avenue, Cambridge, MA 02139, USA
[3]Computational and Systems Biology, Massachusetts Institute of Technology, 77 Massachusetts Avenue, Cambridge, MA 02139, USA
[4]Department of Electrical Engineering and Computer Science, Massachusetts Institute of Technology, 77 Massachusetts Avenue, Cambridge, MA 02139, USA

[*]To whom correspondence should be addressed. Tel: +1 617 253 6039; Email: gifford@mit.edu

1 Supplementary Methods

1.1 Position probability matrices and position weight matrices

We use position probability matrices (PPM) with a DNA alphabet ($\Sigma = \{\mathrm{A}, \mathrm{C}, \mathrm{G}, \mathrm{T}\}$) to represent sequence motifs:

$$\overbrace{\begin{pmatrix} & \mathrm{A} & \mathrm{C} & \mathrm{G} & \mathrm{T} \\ 1 & y_{1,\mathrm{A}} & y_{1,\mathrm{C}} & y_{1,\mathrm{G}} & y_{1,\mathrm{T}} \\ 2 & y_{2,\mathrm{A}} & y_{2,\mathrm{C}} & y_{2,\mathrm{G}} & y_{2,\mathrm{T}} \\ \vdots & \vdots & \vdots & \vdots & \vdots \\ n & y_{n,\mathrm{A}} & y_{n,\mathrm{C}} & y_{n,\mathrm{G}} & y_{n,\mathrm{T}} \end{pmatrix}}^{\text{PPM}} \tag{1}$$

As the name suggests, each cell of a PPM is a probability, the probability of observing a particular nucleotide at a particular position, and each row sums to one, i.e., at each position one of the four nucleotides must be present. We use the notation $\mathrm{PPM}_k(i, j)$ to access the probability of observing the jth nucleotide at the ith position in a specific PPM_k.

These PPMs usually describe experimentally obtained estimates of transcription factor binding sites, but may also describe artificially constructed sequence motifs.

To calculate the likelihood of a sequence given a PPM, we first convert the PPM to a position weight matrix (PWM) by transforming the elements of the PPM to log likelihoods,

$$y'_{i,j} = \log_2 \frac{y_{i,j}}{p_j}, \tag{2}$$

using background sequence probabilities $\boldsymbol{p}$, which are described in the Methods section. The *score* of a particular position in a DNA sequence is then calculated by adding the value of the observed nucleotide at each position in the PWM.

1.2 Motif information content

To calculate the information content of a sequence motif represented as a PPM, we first calculate $\mathrm{U}(i)$, the uncertainty at position i as follows:

$$\mathrm{U}(i) = -\sum_{j \in \Sigma} \mathrm{PPM}(i, j) \times \log_2(\mathrm{PPM}(i, j)). \tag{3}$$

The information content at position i is then defined as follows

$$\mathrm{IC}(i) = t - \mathrm{U}(i), \tag{4}$$

where $t = \log_2(|\Sigma|)$, the total information content per position in bits. In order to obtain MIC, the information content of the entire motif, we add up the individual positions:

$$\mathrm{MIC} = \sum_{i=1}^{n} \mathrm{IC}(i), \tag{5}$$

where n is the motif width in nucleotides (nt), see matrix in 1.

1.3 Relative entropy between motif and background distribution

The information content of a motif is a special case of the relative entropy of a motif where background probabilities $\boldsymbol{p}$ are uniform. Relative entropy, also known as KL divergence, between a motif and the background distribution is calculated per position, similarly to IC:

$$D_{\mathrm{KL}}(i) = \sum_{j \in \Sigma} \mathrm{PPM}(i, j) \times \log_2 \left(\frac{\mathrm{PPM}(i, j)}{p_j} \right), \tag{6}$$

and then summed over positions to obtain the Motif Relative Entropy,

$$\mathrm{MRE} = \sum_{i=1}^{n} D_{\mathrm{KL}}(i). \tag{7}$$

1.4 Relative entropy between two motifs

While the relative entropy between a particular motif, PPM_1, and the background distribution is a way to gauge the learnability of a grammar where the presence of PPM_1 carries information, the relative entropy between two motifs, PPM_1 and PPM_2, is equally useful to assess the learnability of grammars with multiple, semantically distinct sequence elements.

By slightly adjusting the D_{KL} from above, we calculate the KL divergence of position i between two motifs as follows:

$$D_{\mathrm{KL}}(\mathrm{PPM}_1, \mathrm{PPM}_2, i) = \sum_{x \in \Sigma} \mathrm{PPM}_1(i, x) \times \log_2 \left(\frac{\mathrm{PPM}_1(i, x)}{\mathrm{PPM}_2(i, x)} \right). \tag{8}$$

The motif pair relative entropy of PPM_1 relative to PPM_2 is then defined as

$$\mathrm{MPRE}(\mathrm{PPM}_1, \mathrm{PPM}_2) = \sum_{i=1}^{n} D_{\mathrm{KL}}(\mathrm{PPM}_1, \mathrm{PPM}_2, i). \tag{9}$$

To calculate the MPRE between motifs of unequal width, we pad the shorter motifs with *neutral* positions using background probabilities.

Another issue with equation 9 is that it does not capture highly similar but shifted motifs. PPM_1 might be equivalent to PPM_2 shifted by one position and thus considered highly similar, but $\mathrm{MPRE}(\mathrm{PPM}_1, \mathrm{PPM}_2)$ in its current form does not reflect this. To resolve this, we calculate $\mathrm{MPRE}(\mathrm{PPM}_1, \mathrm{PPM}_2)$ for several alignments of PPM_1 and PPM_2 and take the minimum.

1.5 Empirical similarity score between two motifs

The empirical similarity score (ESS) between PPM_1 and PPM_2 is another way to assess the similarity between two motifs and thus the difficulty to distinguish between them. $\mathrm{ESS}(\mathrm{PPM}_1, \mathrm{PPM}_2)$ is calculated by generating k (in this work, $k = 100$) instances of motif 2, flanked on both sides by background sequences of length n_1, where n_1 is the width of PPM_1. All positions of these k sequences are then scored by PWM_1 (the position weight matrix of PPM_1), and the highest score per sequence is returned. $\mathrm{ESS}(\mathrm{PPM}_1, \mathrm{PPM}_2)$ is then the mean of these k scores. ESS motif matrix plots (Supplementary Figure S4 and Supplementary Figure S5) depict adjusted empirical similarity scores, which are shifted by ESS_0 if $\mathrm{ESS}_0 < 0$, where $\mathrm{ESS}_0 = \min_j \mathrm{ESS}(\mathrm{PPM}_i, \mathrm{PPM}_j)$, and normalized such that the self similarity score $\mathrm{ESS}(\mathrm{PPM}_i, \mathrm{PPM}_i) = 1.0$.

Both MPRE and ESS are asymmetric, i.e., $\mathrm{ESS}(\mathrm{PPM}_1, \mathrm{PPM}_2) \neq \mathrm{ESS}(\mathrm{PPM}_2, \mathrm{PPM}_1)$.

1.6 Selection of unambiguous set of HOMER sequence motifs

In order to generate synthetic data sets that are closer to experimentally obtained data sets, we replaced the artificially constructed k-mers used in the insertion probability grammar of Supplementary Figure S2 with transcription factor binding site motifs which were obtained from ChIP-seq assays and curated by HOMER. However, before a collection of experimentally obtained motifs can be used effectively as sequence elements in grammars, degenerate motifs must be excluded. These include motifs with low information content and highly similar motif pairs. If these motifs are used as sequence elements that encode information about the condition y, but either cannot be differentiated from the background distribution or motifs specific to one condition are highly similar to motifs specific to another condition, the conditions are rendered inseparable and learning becomes impossible. This scenario is shown in Supplementary Figure S4A, which depicts the test set ROC curves of a Bayes Optimal Classifier (BOC) for 10 classes of a data set generated by a grammar using 10 randomly selected HOMER motifs as class-specific sequence elements. BOCs in the context of seqgra are used to determine whether the conditions of a grammar are separable in principle, i.e., regardless of data set size and neural network architecture. Instead of neural network models whose weights are adjusted during training, the BOC has access to the data definition and uses the rules and sequence elements specified there directly to classify the examples. If the predictive performance of the BOC is low, as is the case with conditions C_6, C_8, and C_4 shown in Supplementary Figure S4A, the rules associated with those conditions are not specific enough to differentiate between them. And since the rules in this case place a supposedly condition-specific sequence element at a random position in the sequence window, the only explanation is

that these sequence elements are either indistinguishable from background or indistinguishable from each other. The latter is shown in the matrices in Supplementary Figure S4C and Supplementary Figure S4D, which identify the corresponding sequence elements SE_6, SE_8, and SE_4 as most similar to other sequence elements, i.e., lowest KL divergence and highest empirical similarity score, respectively (see Methods for details).

Supplementary Figure S4B shows BOC performance after the most ambiguous motifs were removed, and the corresponding KL divergence and empirical similarity score matrices are shown in Supplementary Figure S4E and F. A collection of experimentally derived sequence motifs will never be completely orthogonal, but the degree of dissimilarity between these 10 were deemed sufficient and all subsequent multi-class classification grammars with 10 classes used these 10 motifs. Supplementary Figure S5 shows the same selection process for a collection of 100 HOMER motifs. All HOMER motifs used in this study are listed in Supplementary Table S1, together with a IUPAC notation of the motif, the motif information content and the KL divergence between the motif and the background distribution (see Methods for details). Motifs used for binary classification tasks are listed in Supplementary Table S2, those for multi-class classification tasks with 10, 20, and 50 classes are listed in Supplementary Tables S3, S4, and S5, respectively.

1.7 seqgra core functionality

1.7.1 Insertion probability test

To showcase the seqgra core pipeline, we used a simple grammar, similar to the one described in example 1 of Figure 1D, but instead of always inserting the class-specific k-mer, we used different insertion probabilities for each class, ranging from 100 % present in examples of class 1, C_1, to 80 % present in C_2, 60 % present in C_3, 40 % present in C_4, 20 % present in C_5, 10 % present in C_6, 5 % present in C_7, and only present in 1 % of C_8 examples. We chose a neural network architecture with two hidden layers, a convolutional layer, followed by a fully connected layer (Supplementary Figure S2A). After the simulation process finished, diagnostic plots were generated, depicting a heatmap of grammar positions for all examples per class (Supplementary Figure S2B). These so-called positional grammar probabilities (i.e., the probability for a specific position to be a grammar position), depicted in the heatmap correspond to the insertion probabilities of the grammar, as expected. Furthermore, the class-specific ROC curves in Supplementary Figure S2C show that the chosen neural network architecture was optimal in terms of predictive performance, with true positive rates of 1.0, 0.8, 0.6, 0.4, 0.2, 0.1, 0.05, and 0.01 (at the zero false positive level) for the classes C_1 to C_8, which are the theoretical upper limits given the insertion probabilities of the underlying grammar. This is also reflected in the precision-recall curves in Supplementary Figure S2D. In panels E to G we show the results of the feature importance evaluators raw gradient, absolute gradient, and Sufficient Input Subsets (see Methods for details). These heatmaps show whether the model's predictions were based on relevant (i.e., grammar) positions and are therefore an indication of the model's ability to recover the underlying grammar of the data set. All three methods suggest high grammar recovery (many dark green positions, few dark red positions).

Supplementary Figure S2 covered the results obtained from a single seqgra call, evaluating one neural network model trained on one synthetic data set, but most seqgra analyses compare various different architectures across a range of data sets (of different grammar complexities and sizes). For these situations, we provide a suite of convenient commands that streamline these analyses and provide a schematic description of their inputs and outputs in Supplementary Figure S1.

1.7.2 Protein grammar and model test

While the previous section described a seqgra analysis with synthetic DNA sequences and models trained on DNA, here we demonstrate seqgra's ability to synthesize and subsequently train and evaluate models on protein sequences.

For this example we prepared a binary classification grammar with protein sequences of class 1 (C_1) containing phosphorylation sites of the kinase PLK1 and class 2 (C_2) protein sequences containing sites of the kinase CDK1. The grammar was defined using simplified position probability matrices of the phospho-acceptor sites of the respective kinase (sequence logos are shown in Supplementary Figure S3A), obtained from `https://scansite4.mit.edu`. The predictive model was implemented in PyTorch, consisting of a minimal neural network architecture with one convolutional layer with 10 filters, each 5 amino acids wide, global max pooling, and a fully connected layer with 5 units. Heatmap of grammar

positions (Supplementary Figure S3B), ROC (Supplementary Figure S3C) and PR curves (Supplementary Figure S3D), as well as the results of feature importance evaluators (Supplementary Figure S3E-F) are similar to the DNA sequence example above. With ROC AUC and PR AUC of both approx. 0.99, the model's predictive performance was near-perfect, which was expected given the simplistic grammar. However, the feature importance evaluators showed that the model failed to learn the slightly more difficult PLK1 motif (C_1) and instead learned the CDK1 motif from C_2 examples. Learning C_1 specifically was not necessary for the task with two mutually exclusive classes.

2 Supplementary Figures

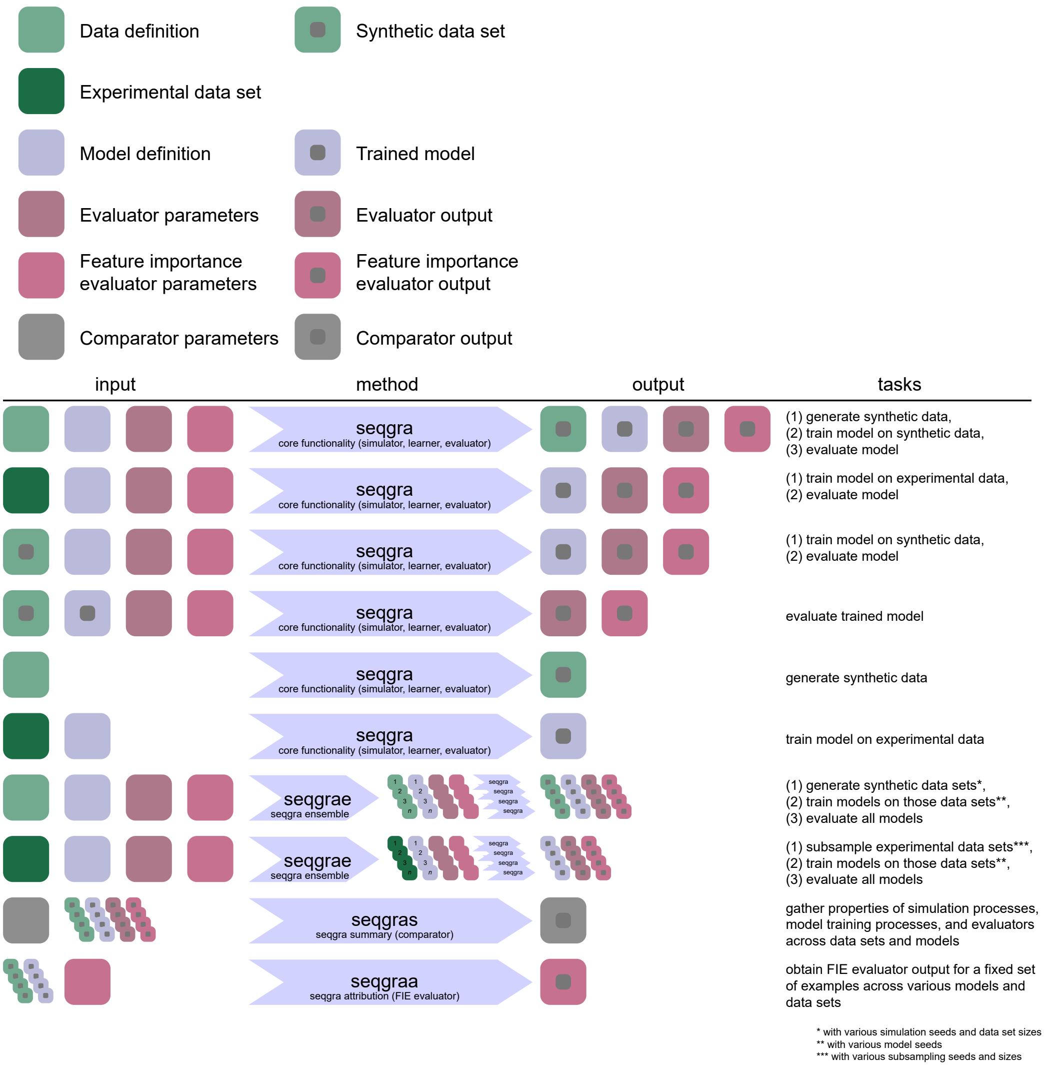

Supplementary Figure S1: Schematic of common use cases for seqgra command line interface. The seqgra package contains four commands, `seqgra`, `seqgrae`, `seqgras`, and `seqgraa`. `seqgra` contains the core functionality of (1) generating synthetic data using the *Simulator* component, (2) training models on either synthetic or experimental data using various *Learner* components, and (3) evaluating the model using various *Evaluator* components. `seqgrae`, short for seqgra ensemble, is a convenient way to generate multiple synthetic data sets with various data set sizes and simulation seeds and train models on them using a range of model seeds. `seqgras`, short for seqgra summary, is a tool to gather properties and metrics across a number of data sets and models and compare them using *Comparator* components. `seqgraa`, short for seqgra attribution, runs feature importance evaluators on a number of trained models, using the same set of examples each time.

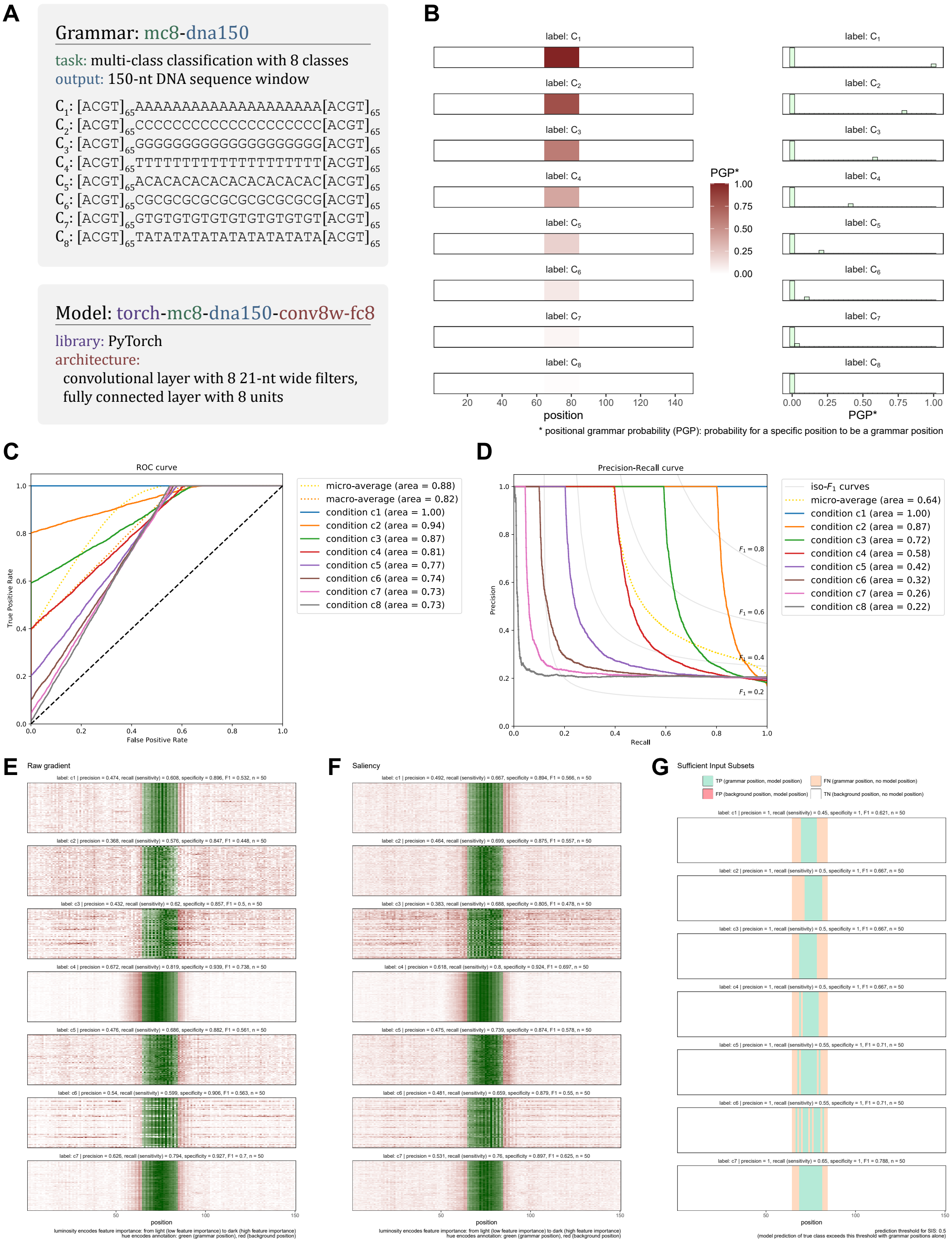

Supplementary Figure S2: Insertion probability test. (**A**) Grammar and model description. (**B**) Grammar position heatmap (on the left) depicting the probability of grammar annotation for all positions (1 - 150) and all classes (C_1 to C_8). (**C**) Test set ROC curve of classifier trained on synthetic data depicts class-specific true positive rates that mirror insertion probabilities, as expected. (**D**) Test set PR curve of same classifier, class-specific curves mirror insertion probabilities. (**E**) Raw gradient feature importance for classes C_1 to C_7 (classifier did not correctly predict class C_8). The x-axis is the position in the sequence window, the y-axis are randomly drawn examples with that class label. Dark green areas are grammar positions with high feature importance (desired), dark red areas are background positions with high feature importance (undesired). (**F**) Same as panel E, for absolute gradient (saliency) feature importance. (**G**) Same as panel E, for Sufficient Input Subsets (SIS) feature importance. The only difference is that SIS is an inherently discrete measure of feature importance, either positions are part of a sufficient input subset or not.

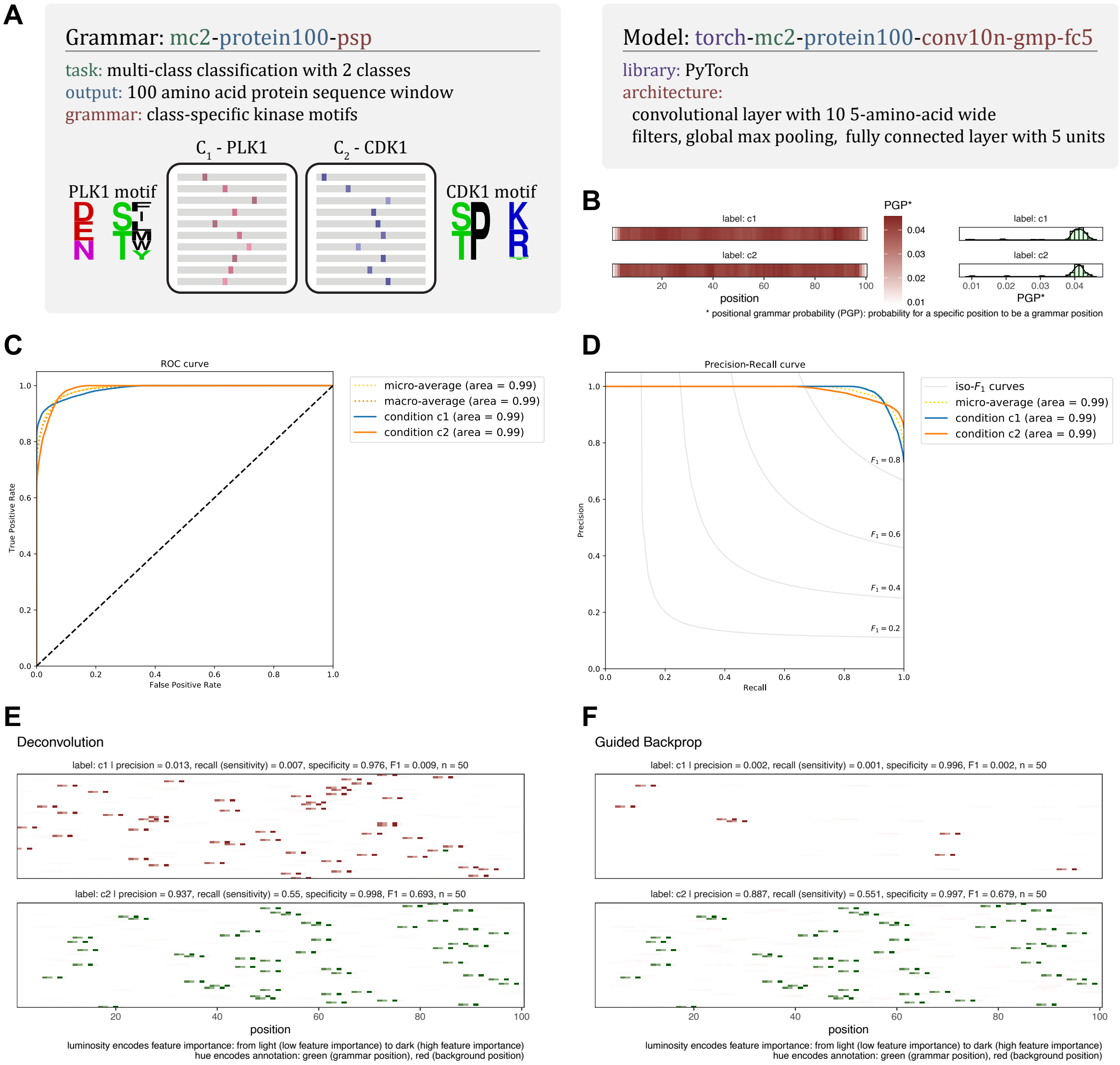

Supplementary Figure S3: Protein grammar and model test. (**A**) Grammar and model description. (**B**) Grammar position heatmap (on the left) depicting the probability of grammar annotation for all positions (1 - 100) and all classes (C_1 and C_2). (**C**) Test set ROC curve of classifier trained on synthetic data depicts class-specific true positive rates. (**D**) Test set PR curve of same classifier. (**E**) Deconvolution feature importance for classes C_1 and C_2 shows near perfect overlap with grammar positions for class C_2 (CDK1 kinase motif) and poor overlap for class C_1 (PLK1 kinase motif). The x-axis is the position in the sequence window, the y-axis are randomly drawn examples with that class label. Dark green areas are grammar positions with high feature importance (desired), dark red areas are background positions with high feature importance (undesired). (**F**) Same as panel E, for guided backpropagation feature importance method.

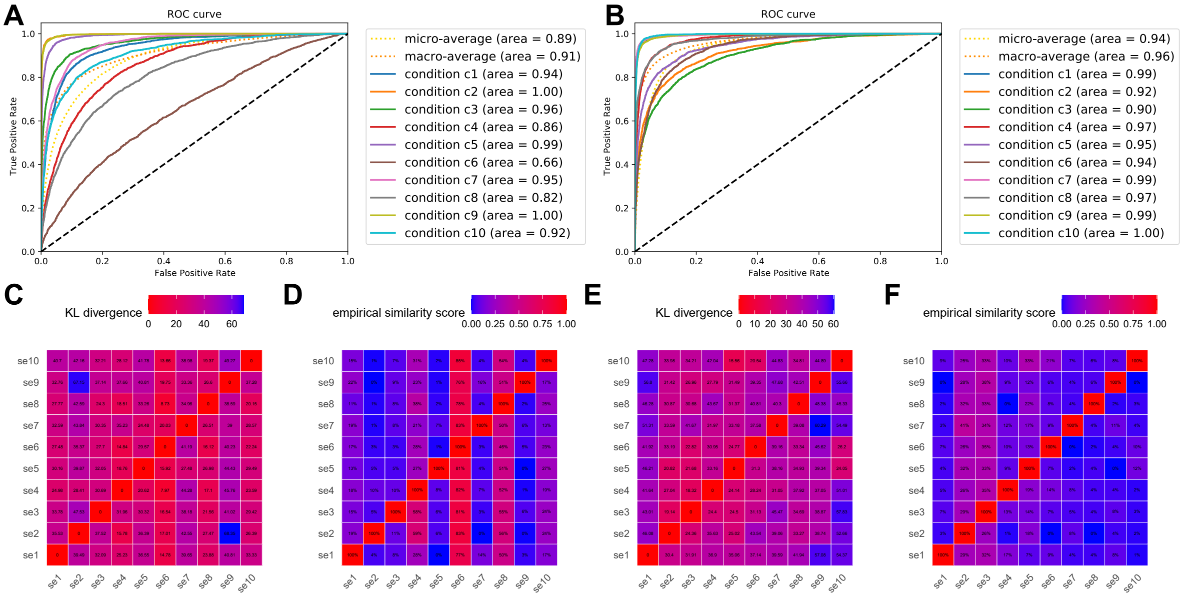

Supplementary Figure S4: Selection of sequence motifs for simulation grammars. (**A**) ROC curve of Bayes Optimal Classifier on multi-class classification task with 10 classes, prior to filtering out ambiguous sequence motifs. (**B**) Same as panel A, after ambiguous sequence motifs were removed. (**C**) KL divergence matrix of 10 sequence motifs, prior to filtering. (**D**) Empirical similarity score matrix of 10 sequence motifs, prior to filtering. (**E**) Same as panel C, after removing ambiguous motifs. (**F**) Same as panel D, after removing ambiguous motifs.

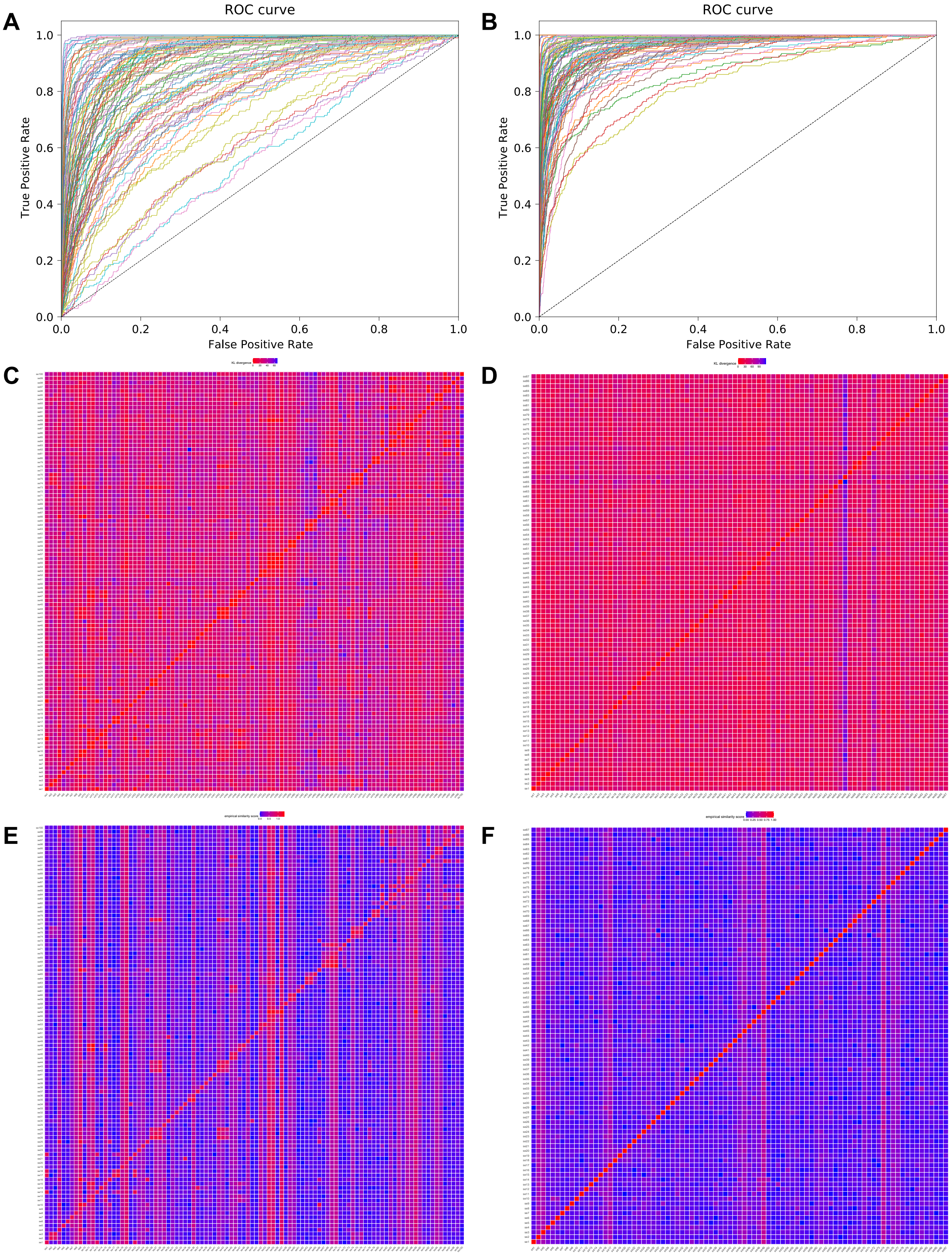

Supplementary Figure S5: Selection of sequence motifs for MC100 simulation grammars. (**A**) ROC curve of Bayes Optimal Classifier on multi-class classification task with 100 classes, prior to filtering out ambiguous sequence motifs. (**B**) Same as panel A, after ambiguous sequence motifs were removed. (**C**) KL divergence matrix of 100 sequence motifs, prior to filtering. (**D**) Same as panel C, after removing ambiguous motifs. (**E**) Empirical similarity score matrix of 100 sequence motifs, prior to filtering. (**F**) Same as panel D, after removing ambiguous motifs.

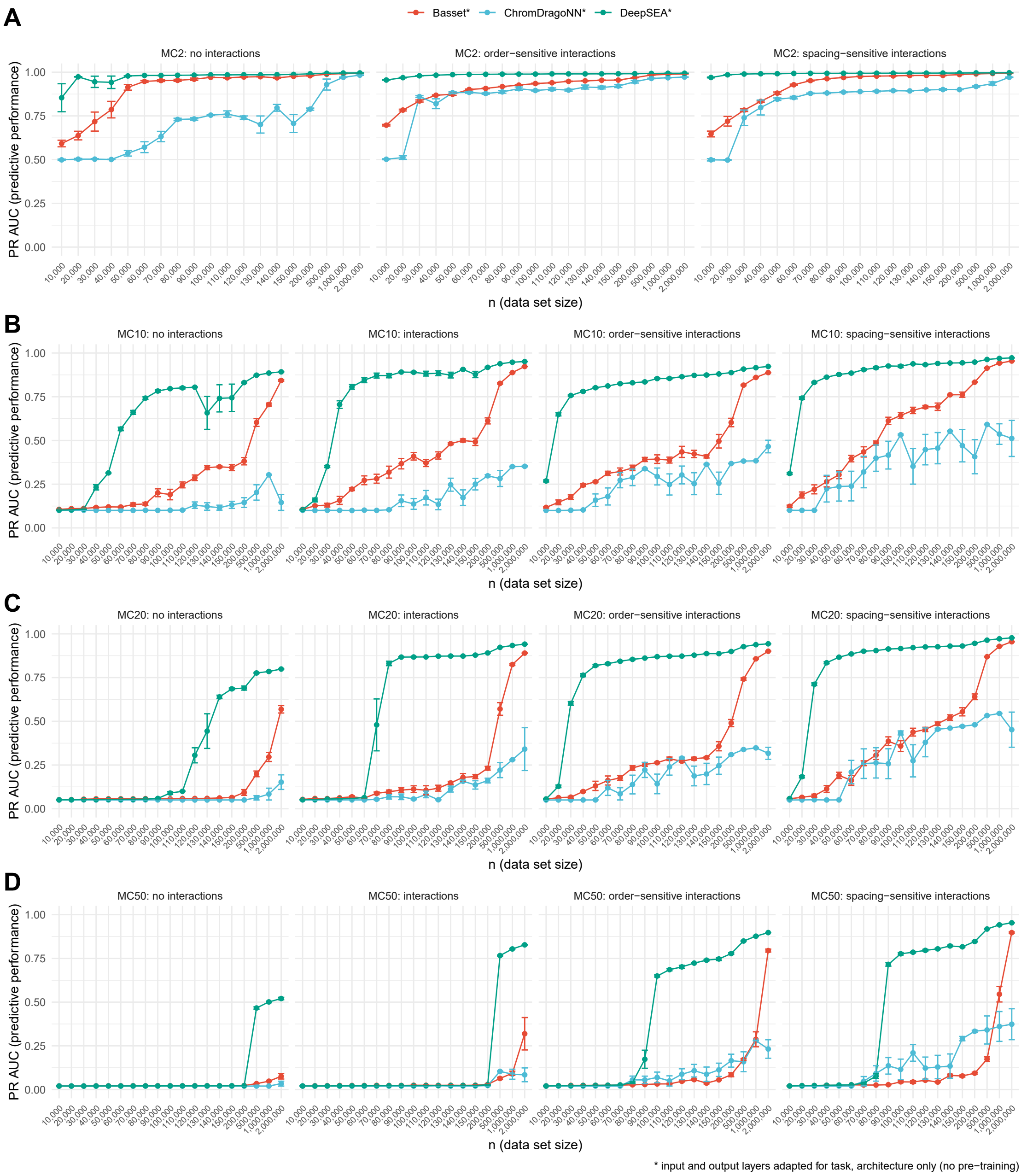

Supplementary Figure S6: Comparison of neural network architectures Basset, ChromDragoNN, and DeepSEA. (**A**) Predictive performance on binary classification tasks of grammars with class-specific HOMER motifs (left), class-specific order of HOMER motifs (middle), and class-specific spacing of HOMER motifs. All architectures were trained on data sets ranging in size from 10,000 examples to 2,000,000 examples. Error bars are standard errors of five models trained on the same grammar, using five different simulation seeds. (**B**) Same as panel A, for multi-class classification tasks with 10 classes. The second plot from the left shows the predictive performance of models trained on data sets with class-specific interactions of HOMER motifs. (**C**) Same as panel B, for multi-class classification tasks with 20 classes. (**D**) Same as panel B, for multi-class classification tasks with 50 classes.

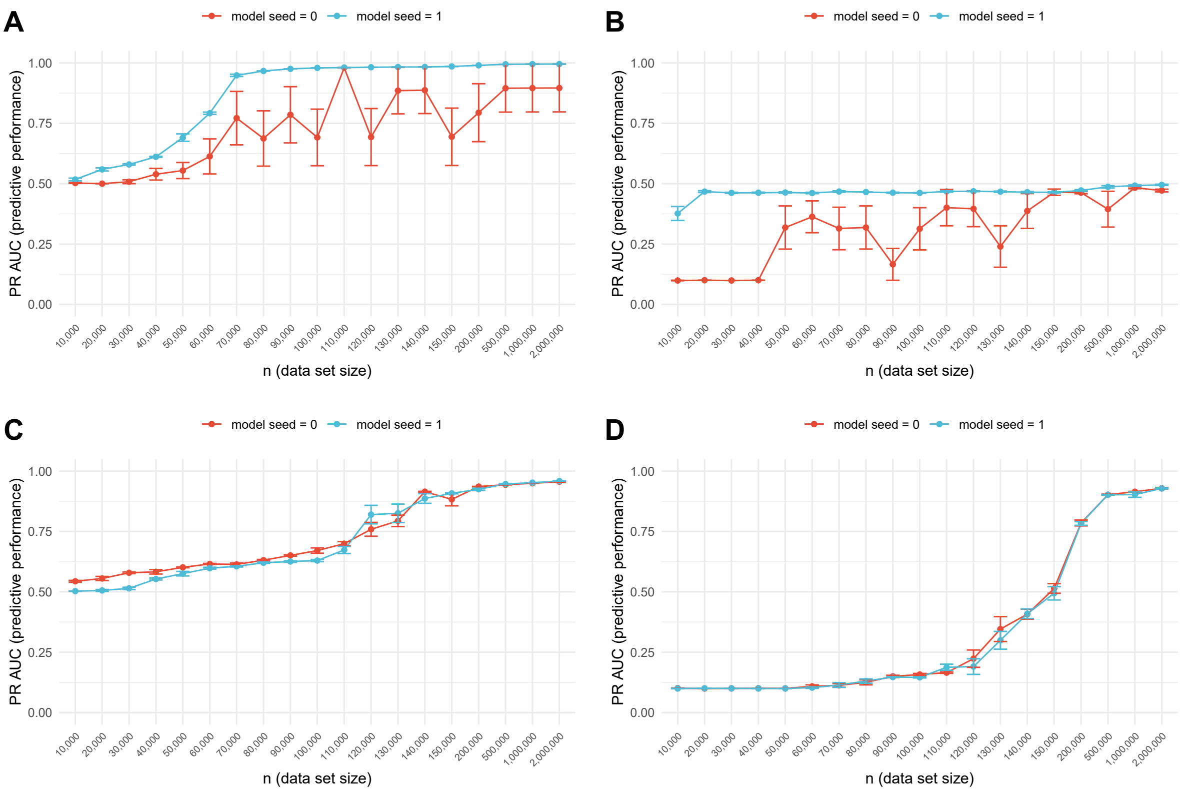

Supplementary Figure S7: PyTorch and TensorFlow affected by random seed induced instability. (**A**) Shown are test set PR AUCs of a PyTorch neural network architecture with two hidden layers, a convolutional layer with 10 21-nt wide filters followed by a dense layer with 5 units, trained on binary classification data sets using HOMER motifs without interactions. This PyTorch neural network architecture exhibits an unusual variability in PR AUC when trained with a random seed of zero. (**B**) Shown are test set PR AUCs of a TensorFlow neural network architecture with a convolutional layer with 10 21-nt wide filters, a global max pooling operation, and a dense layer with 10 units, trained on multi-class classification data sets with 10 classes using HOMER motifs with spacing-sensitive interactions. This TensorFlow neural network architecture exhibits an unusual variability in PR AUC when trained with a random seed of zero. (**C**) Unlike panel A, this PyTorch neural network architecture (convolutional layer with 10 11-nt wide filters followed by a dense layer with 5 units), which was trained on the same data sets as the one in panel A, does not exhibit unusually high variability of PR AUCs when trained with a random seed of zero. (**D**) Similarly, this TensorFlow neural network architecture (convolutional layer with 10 21-nt wide filters followed by a dense layer with 10 units), which was trained on the same data sets as the one in panel B, also does not exhibit unusually high variability of PR AUCs when trained with a random seed of zero.

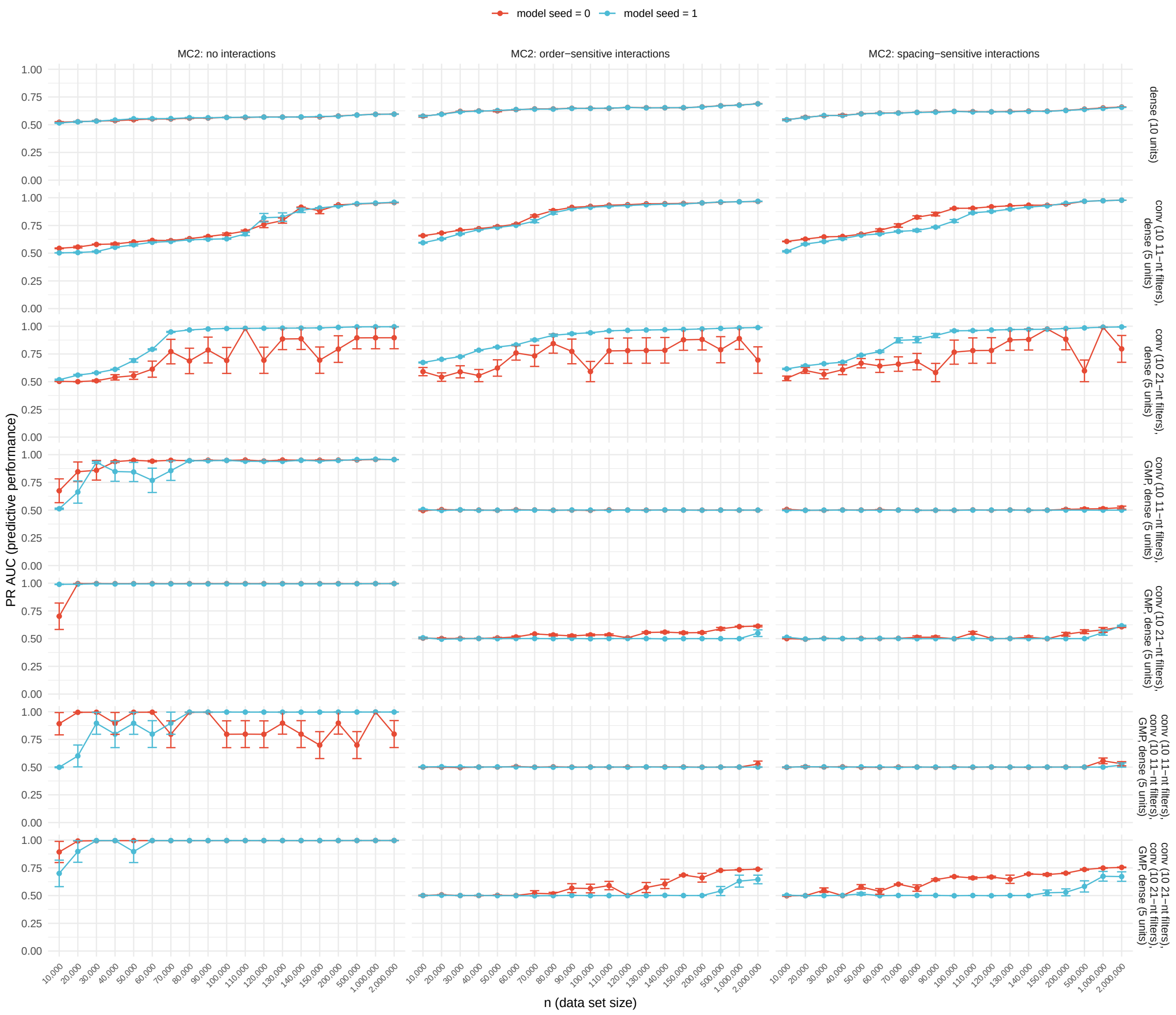

Supplementary Figure S8: PyTorch models trained with random seed 0 suffer from grammar-dependent and architecture-dependent instability. Not all grammar-architecture combinations are affected.

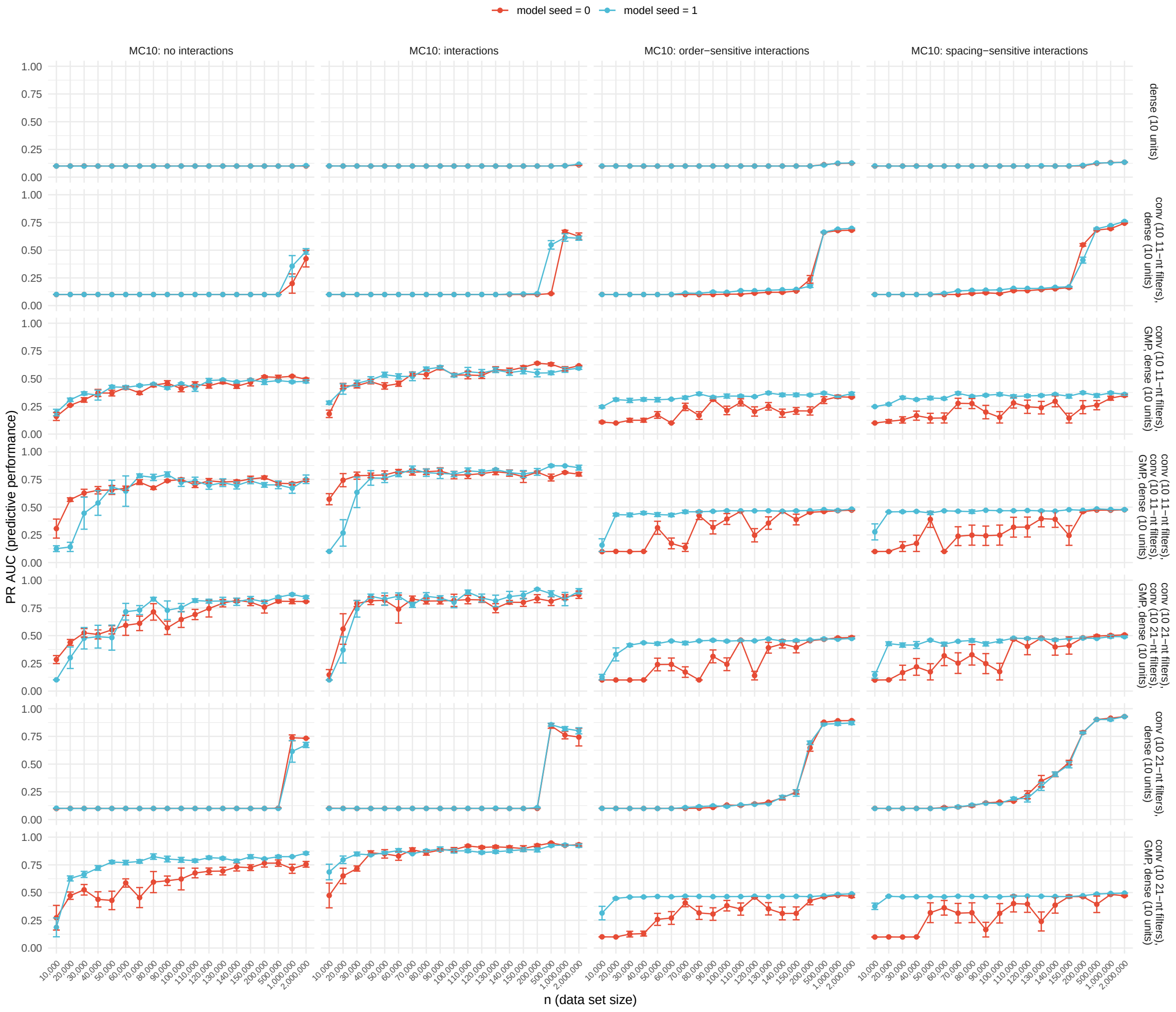

Supplementary Figure S9: TensorFlow models trained with random seed 0 suffer from grammar-dependent and architecture-dependent instability. Not all grammar-architecture combinations are affected.

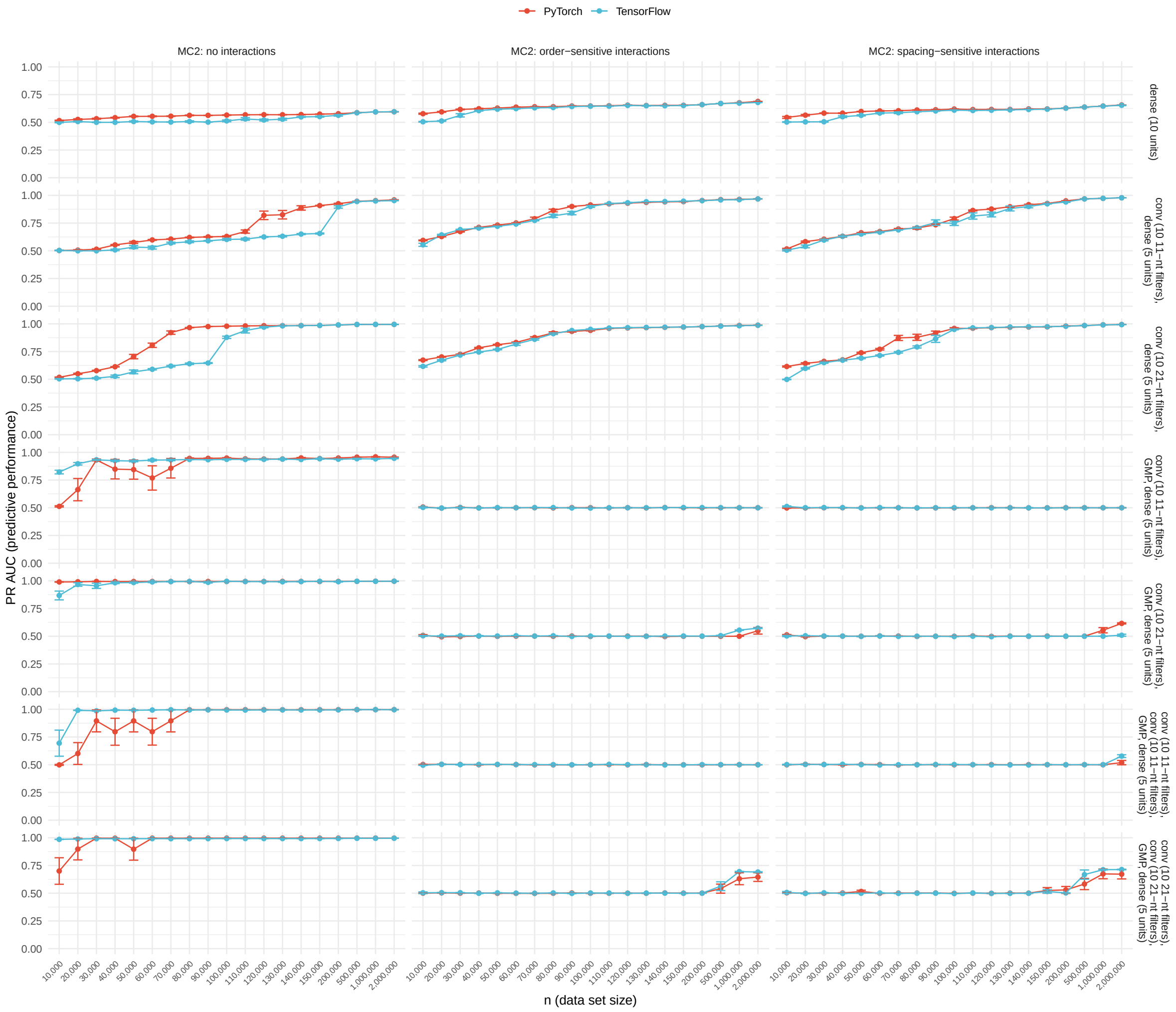

Supplementary Figure S10: Comparison of PyTorch and TensorFlow architectures trained on binary classification data sets. When comparing an equivalent neural network architecture, trained on the same data set, test set PR AUCs between models implemented and trained with deep learning libraries PyTorch and TensorFlow are similar. Shown here are comparisons across three grammars, 19 data set sizes, and seven neural network architectures.

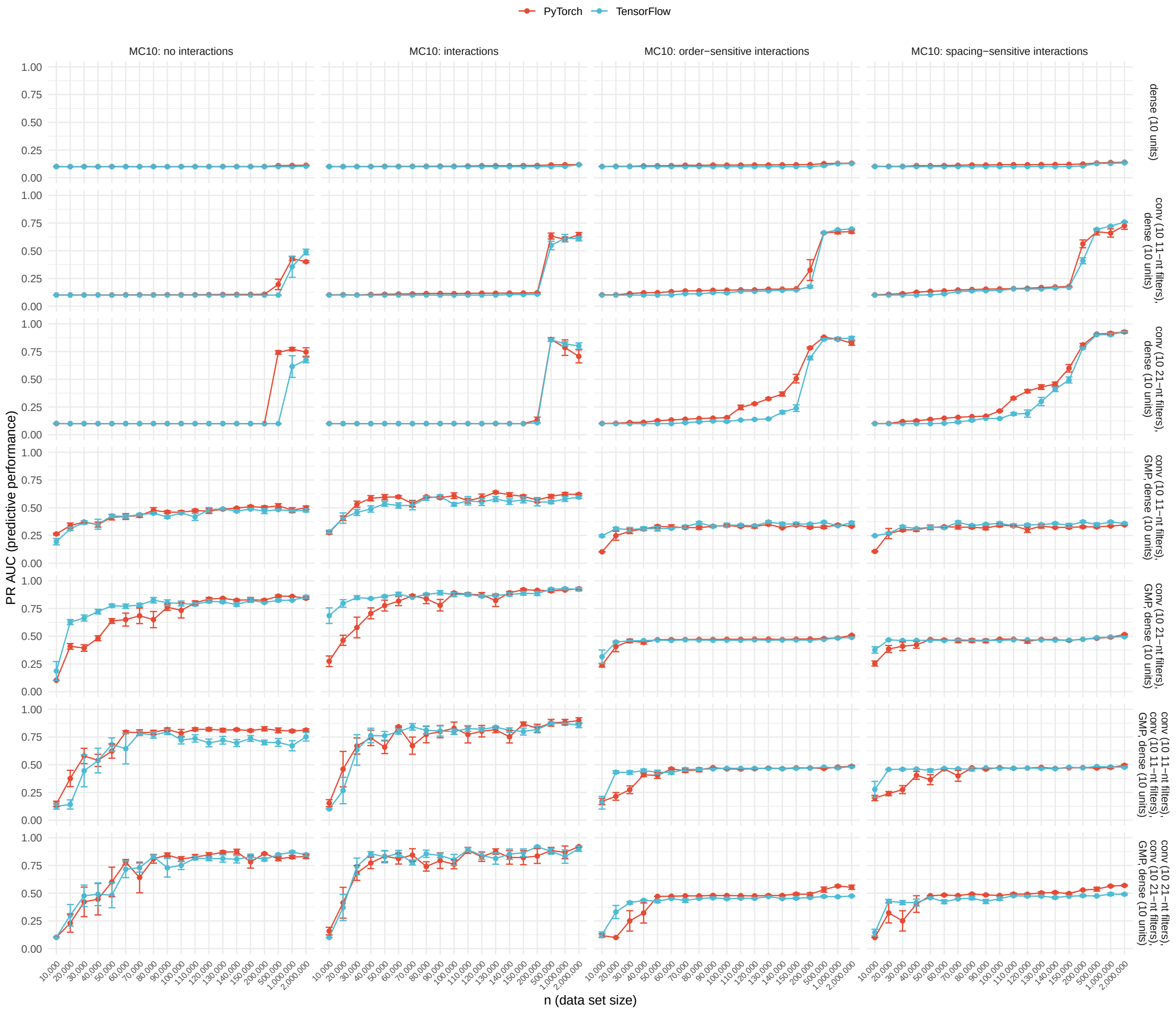

Supplementary Figure S11: Comparison of PyTorch and TensorFlow architectures trained on multi-class classification data sets with 10 classes. When comparing an equivalent neural network architecture, trained on the same data set, test set PR AUCs between models implemented and trained with deep learning libraries PyTorch and TensorFlow are similar. Shown here are comparisons across four grammars, 19 data set sizes, and seven neural network architectures.

3 Supplementary Tables

ID	Motif	IUPAC notation	Width (in nt)	MIC	$D_{\mathrm{KL}}(\cdot)$
se1	FOXA1:AR	AGTAAACAAAAAAGAACANA	20	18.9	17.4
se2	Bcl11a	TYTGACCASWRG	12	11.3	11.8
		MC2 grammar motifs above			
se3	Brachyury	ANTTMRCASBNNNGTGYKAAN	21	11.5	11.6
se4	CEBP:CEBP	NTNATGCAAYMNNHTGMAAY	20	14.8	14.3
se5	Chop	ATTGCATCAT	10	13.2	12.7
se6	CHR	CGGTTTCAAA	10	12.6	11.8
se7	CTCF-SatelliteElement	TGCAGTTCCAANAGTGGCCA	20	18.8	19.6
se8	Mouse Recombination Hotspot	ACTYKNATTCGTGNTACTTC	20	15.3	14.9
se9	RAR:RXR	RGGTCADNNAGAGGTCAV	18	16.3	17.3
se10	DUX	BCWGATTCAATCAAN	15	17.9	16.9
		MC10 grammar motifs above			
se11	E2F7	VDTTTCCCGCCA	12	13.4	14.6
se12	EBNA1	GGYAGCAYDTGCTDCCCNNN	20	18.1	19.2
se13	ERE	AAGGTCACNGTGACC	15	14.3	15.2
se14	ETS:E-box	AGGAAACAGCTG	12	17.3	17.6
se15	EWS:ERG-fusion	ATTTCCTGTN	10	13.7	13.5
se16	Foxh1	NNTGTGGATTSS	12	11.3	11.1
se17	FXR	AGGTCANTGACCTN	14	12.3	13.2
se18	GATA3	AGATGKDGAGATAAG	15	17.3	16.5
se19	GATA3	AGATSTNDNNDSAGATAASN	20	16.9	16.3
se20	GATA	NAGATWNBNATCTNN	15	14.0	13.3
		MC20 grammar motifs above			
se21	GATA:SCL	CGGCTGCNGNNNNCAGATAA	20	15.4	15.9
se22	Gfi1b	AAATCACTGC	10	13.9	13.8
se23	GRHL2	AAACYKGTTWDACMRGTTTB	20	13.5	13.4
se24	Hand2	TGACANARRCCAGRC	15	13.2	13.6
se25	HINFP	TWVGGTCCGC	10	11.7	13.2
se26	HOXB13	TTTTATKRGG	10	13.5	12.6
se27	LRF	AAGACCCYYN	10	11.2	12.5
se28	LXRE	GGGTTACTANAGGTCA	16	17.5	17.9
se29	NF1:FOXA1	NNTGTTTATTTTGGCA	16	17.3	16.7
se30	NFAT:AP1	GAATGGAAAAAATGAGTCAT	20	15.5	15.1
se31	NFAT	ATTTTCCATT	10	13.1	12.5
se32	NFY	AGCCAATCGG	10	13.3	13.8
se33	Nur77	TGACCTTTNCNT	12	15.1	14.8
se34	Oct2	ATATGCAAAT	10	15.3	14.1
se35	Oct4:Sox17	CCATTGTATGCAAAT	15	15.9	15.0
se36	OCT4-SOX2-TCF-NANOG	ATTTGCATAACAATG	15	16.4	14.9
se37	p53	ACATGCCCGGGCAT	14	16.7	18.2
se38	PAX3:FKHR-fusion	ACCGTGACTAATTNN	15	14.6	14.1

Continued on next page

Supplementary Table S1 – *Continued from previous page*

**ID**	**Motif**	**IUPAC notation**	**Width (in nt)**	**MIC**	$D_{\text{KL}}(\cdot)$
se39	PAX5	`GTCACGCTCNCTGA`	14	15.1	16.3
se40	PAX6	`NGTGTTCAVTSAAGCGKAAA`	20	13.9	14.3
se41	Pax7	`NTAATTDGCYAATTANNWWD`	20	16.0	13.9
se42	Pax7	`TAATCAATTA`	10	16.3	14.6
se43	Pax8	`GTCATGCHTGRCTGS`	15	13.4	14.6
se44	Pitx1:Ebox	`YTAATTRAWWCCAGATGT`	18	12.7	11.8
se45	PRDM10	`TGGTACATTCCA`	12	11.9	12.3
se46	PRDM14	`AGGTCTCTAACC`	12	13.7	14.0
se47	PRDM15	`YCCDNTCCAGGTTTT`	15	13.2	13.7
se48	PRDM9	`ADGGYAGYAGCATCT`	15	12.8	13.1
se49	PSE	`WAVTCACCMTAASYDAAAAG`	20	10.6	10.3
se50	RBPJ:Ebox	`GGGRAARRGRMCAGMTG`	17	14.3	15.2

Supplementary Table S1: Homer transcription factor motifs for multi-class classification tasks with 2, 10, 20, and 50 classes (MC2-MC50): These motifs are used for grammars without interactions, with interactions, with interactions with order constraints, and with interactions with spacing constraints. The columns (from left to right) contain the seqgra-internal sequence element ID, the motif name (name of the transcription factor or complex), a summary of the motif in IUPAC notation, the width of the motif in nucleotides, the motif information content (MIC), and the KL divergence between the motif and the background sequence (using the human genomic nucleotide distribution).

**ID**	**Motif**	**min** $D_{\text{KL}}(\cdot,\cdot)$	**max ESS**$(\cdot,\cdot)$
se1	FOXA1:AR	46.1	0 %
se2	Bcl11a	30.4	33 %

Supplementary Table S2: Homer transcription factor motifs for binary classification tasks (MC2): These motifs are used for MC2 grammars without interactions, with interactions, with interactions with order constraints, and with interactions with spacing constraints. The columns (from left to right) contain the seqgra-internal sequence element ID, the motif name (name of the transcription factor or complex), the minimum KL divergence between the motif and the other motif in this grammar, and the maximum adjusted empirical similarity score (ESS) between the motif and the other motif in this grammar.

ID	Motif	min $D_{\mathrm{KL}}(\cdot,\cdot)$	max ESS$(\cdot,\cdot)$
se1	FOXA1:AR	41.6	9 %
se2	Bcl11a	19.1	41 %
se3	Brachyury	18.3	38 %
se4	CEBP:CEBP	24.4	17 %
se5	Chop	15.6	33 %
se6	CHR	20.5	21 %
se7	CTCF-SatelliteElement	31.1	8 %
se8	Mouse Recombination Hotspot	33.3	6 %
se9	RAR:RXR	37.0	11 %
se10	DUX	24.1	12 %

Supplementary Table S3: Homer transcription factor motifs for multi-class classification tasks with 10 classes (MC10): These motifs are used for MC10 grammars without interactions, with interactions, with interactions with order constraints, and with interactions with spacing constraints. The columns (from left to right) contain the seqgra-internal sequence element ID, the motif name (name of the transcription factor or complex), the minimum KL divergence between the motif and the other 9 motifs in this grammar, and the maximum adjusted empirical similarity score (ESS) between the motif and the other 9 motif in this grammar.

ID	Motif	min $D_{\mathrm{KL}}(\cdot,\cdot)$	max ESS$(\cdot,\cdot)$
se1	FOXA1:AR	39.0	10 %
se2	Bcl11a	18.0	40 %
se3	Brachyury	18.3	35 %
se4	CEBP:CEBP	24.4	13 %
se5	Chop	15.6	32 %
se6	CHR	20.5	19 %
se7	CTCF-SatelliteElement	31.1	10 %
se8	Mouse Recombination Hotspot	24.3	9 %
se9	RAR:RXR	35.1	9 %
se10	DUX	24.1	14 %
se11	E2F7	20.1	15 %
se12	EBNA1	35.0	13 %
se13	ERE	16.8	18 %
se14	ETS:E-box	33.1	17 %
se15	EWS:ERG-fusion	20.5	20 %
se16	Foxh1	27.9	30 %
se17	FXR	16.8	35 %
se18	GATA3	30.7	21 %
se19	GATA3	31.2	20 %
se20	GATA	31.7	15 %

Supplementary Table S4: Homer transcription factor motifs for multi-class classification tasks with 20 classes (MC20): These motifs are used for MC20 grammars without interactions, with interactions, with interactions with order constraints, and with interactions with spacing constraints. The columns (from left to right) contain the seqgra-internal sequence element ID, the motif name (name of the transcription factor or complex), the minimum KL divergence between the motif and the other 19 motifs in this grammar, and the maximum adjusted empirical similarity score (ESS) between the motif and the other 19 motif in this grammar.

ID	Motif	min $D_{\mathrm{KL}}(\cdot,\cdot)$	max ESS$(\cdot,\cdot)$
se1	FOXA1:AR	32.7	15 %
se2	Bcl11a	12	45 %
se3	Brachyury	18.3	39 %
se4	CEBP:CEBP	23.0	18 %
se5	Chop	15.6	35 %
se6	CHR	17.6	26 %
se7	CTCF-SatelliteElement	31.1	9 %
se8	Mouse Recombination Hotspot	24.2	10 %
se9	RAR:RXR	22.3	28 %
se10	DUX	24.1	23 %
se11	E2F7	20.1	15 %
se12	EBNA1	35.0	12 %
se13	ERE	16.8	20 %
se14	ETS:E-box	26.9	16 %
se15	EWS:ERG-fusion	17.9	18 %
se16	Foxh1	20.7	36 %
se17	FXR	13.2	38 %
se18	GATA3	28.1	18 %
se19	GATA3	31.2	20 %
se20	GATA	27.4	20 %
se21	GATA:SCL	19.5	14 %
se22	Gfi1b	23.1	14 %
se23	GRHL2	21.2	19 %
se24	Hand2	15.2	30 %
se25	HINFP	25.6	23 %
se26	HOXB13	25.6	14 %
se27	LRF	23.2	20 %
se28	LXRE	20.0	28 %
se29	NF1:FOXA1	29.1	13 %
se30	NFAT:AP1	19.1	17 %
se31	NFAT	17.2	20 %
se32	NFY	19.8	25 %
se33	Nur77	27.6	18 %
se34	Oct2	17.1	29 %
se35	Oct4:Sox17	22.6	15 %
se36	OCT4-SOX2-TCF-NANOG	17.9	14 %
se37	p53	27.3	10 %
se38	PAX3:FKHR-fusion	19.7	17 %
se39	PAX5	20.8	13 %
se40	PAX6	23.6	14 %
se41	Pax7	21.7	23 %
se42	Pax7	20.7	25 %
se43	Pax8	25.4	18 %

Continued on next page

Supplementary Table S5 – *Continued from previous page*

**ID**	**Motif**	**min $D_{\mathrm{KL}}(\cdot,\cdot)$**	**max ESS$(\cdot,\cdot)$**
se44	Pitx1:Ebox	14.1	33 %
se45	PRDM10	14.5	33 %
se46	PRDM14	18.4	17 %
se47	PRDM15	23.0	18 %
se48	PRDM9	17.9	34 %
se49	PSE	15.0	44 %
se50	RBPJ:Ebox	17.8	24 %

Supplementary Table S5: Homer transcription factor motifs for multi-class classification tasks with 50 classes (MC50): These motifs are used for MC50 grammars without interactions, with interactions, with interactions with order constraints, and with interactions with spacing constraints. The columns (from left to right) contain the seqgra-internal sequence element ID, the motif name (name of the transcription factor or complex), the minimum KL divergence between the motif and the other 49 motifs in this grammar, and the maximum adjusted empirical similarity score (ESS) between the motif and the other 49 motif in this grammar.
